# Case Report of Incarcerated Gastric Volvulus and Splenic Herniation in Undiagnosed Congenital Diaphragmatic Hernia in an Infant

**DOI:** 10.21980/J8VD27

**Published:** 2025-07-31

**Authors:** Kate R Gelman, Torren A Kalaskey, Federico G. Seifarth

**Affiliations:** * West Virginia University School of Medicine, Morgantown, WV; ^ WVU Medicine Children’s Hospital, Division of Pediatric Surgery, Morgantown, WV

## Abstract

Congenital diaphragmatic hernia is usually diagnosed prenatally and surgically repaired within the first few days of life. It is very rarely associated with acute gastric volvulus, an uncommon surgical emergency in children. A male infant with atypical presentation of acute gastric volvulus was diagnosed and treated by a swift-acting team across two medical centers. The patient presented with generalized abdominal distention and non-productive retching. Upper gastrointestinal series diagnosed acute gastric volvulus. During laparoscopic intervention, the volvulized stomach and the spleen were identified within an undiagnosed congenital diaphragmatic hernia. Corrective surgery was performed, and the patient tolerated a regular diet within days and continues to do well. Given the emergent nature of acute gastric volvulus in children, a high index of suspicion is warranted to quickly initiate potentially life-saving diagnostics and treatment.

**Topics:**

Acute gastric volvulus, diaphragmatic hernia, pediatric.

**Figure:**
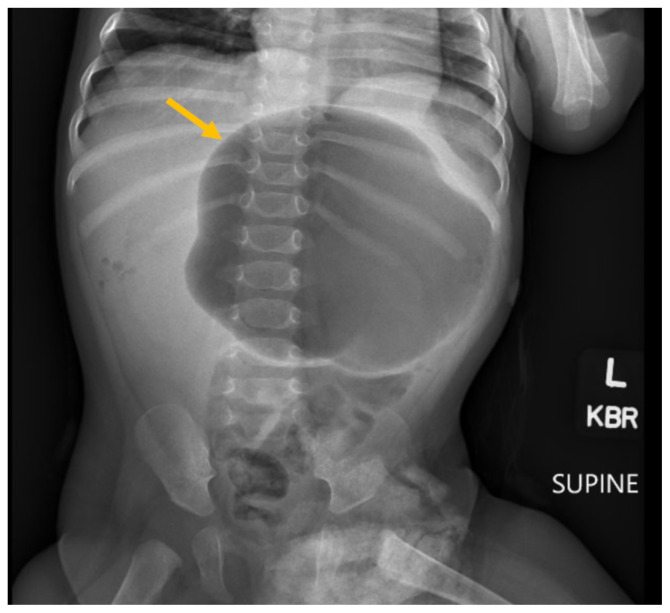


**Figure:**
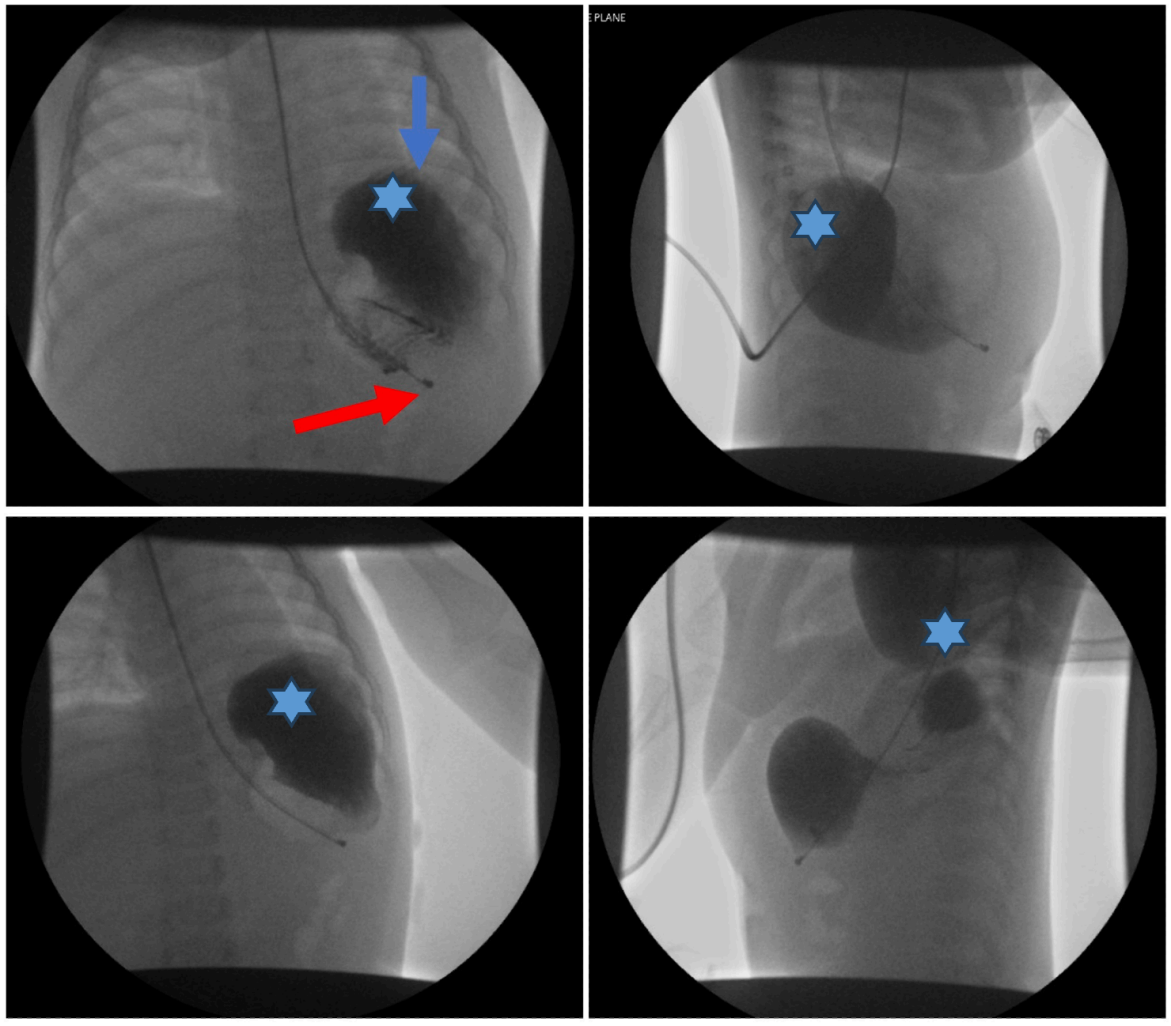


**Figure:**
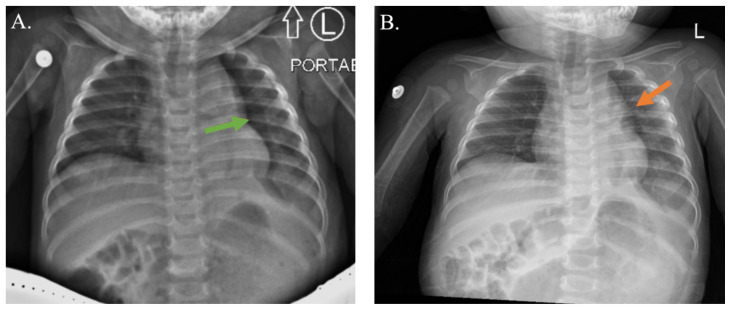


**Figure:**
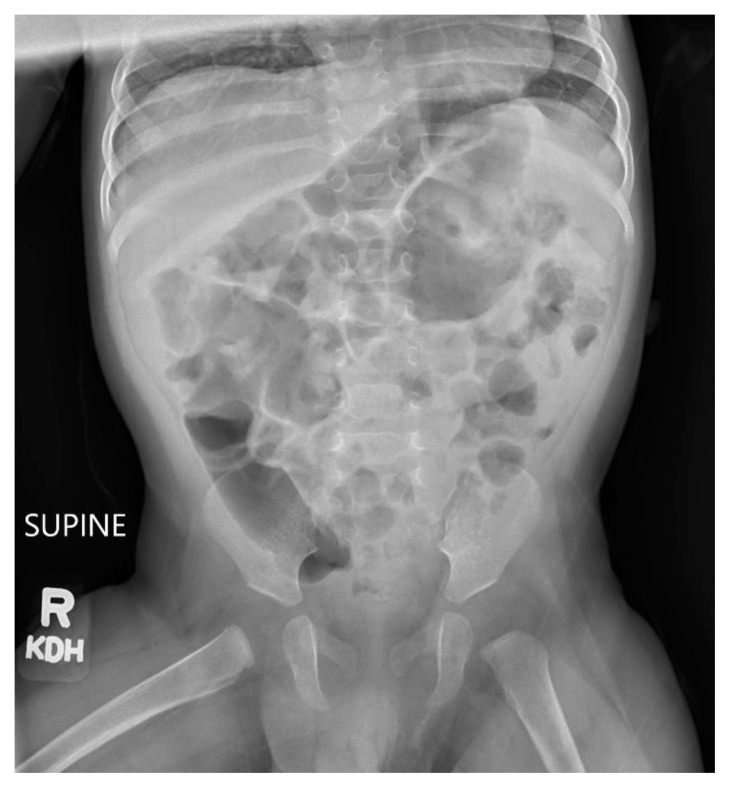


## Brief introduction

Acute gastric volvulus (AGV) is a rare surgical emergency in which the stomach rotates more than 180 degrees along its transverse or longitudinal axis. As shown in the figure, organoaxial volvulus refers to rotation of the stomach around its longitudinal axis (A), while mesenteroaxial volvulus refers to rotation around the mesenteric axis perpendicular to the longitudinal axis (B).

**Figure:**
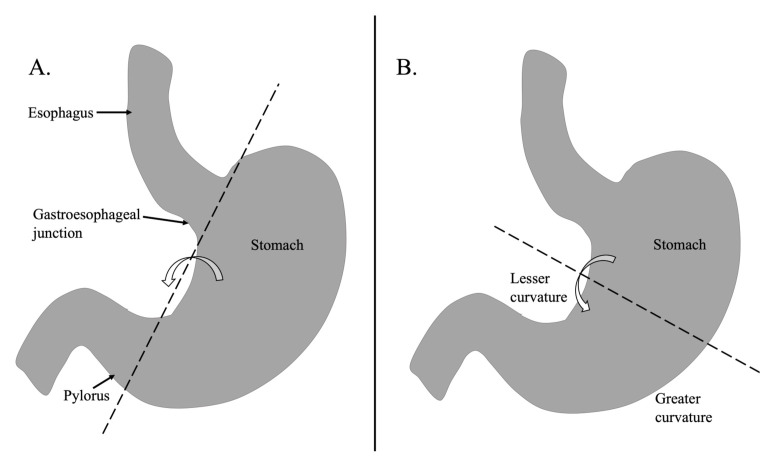


Gastric volvulus in children is usually due to incomplete formation of one of the four ligaments that normally anchor the stomach to the abdominal cavity. The schematic drawing shows the four ligaments anchoring the stomach to prevent rotation (volvulus). The gastrophrenic ligament (A), gastrosplenic ligament (B), and gastrocolic ligament (C) are part of the greater omentum. The hepatogastric ligament (D) is part of the lesser omentum.

**Figure:**
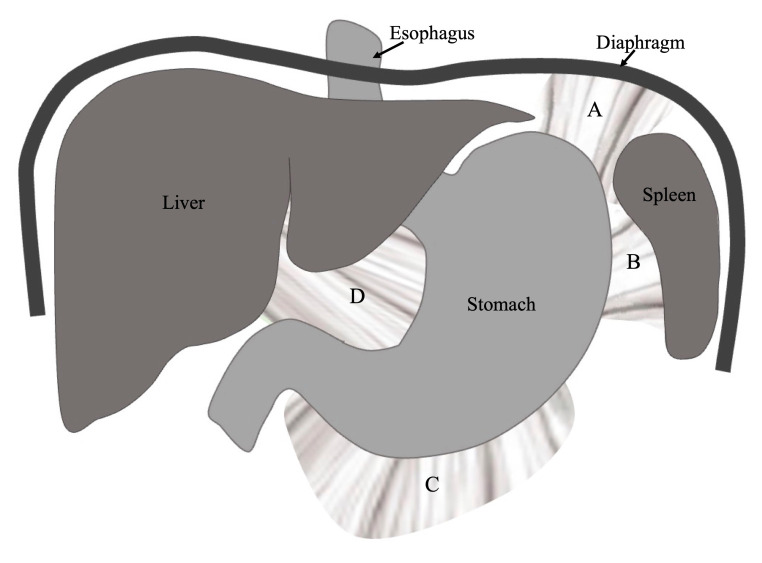


Between 2008 and 2017, only 125 cases of AGV were reported in children under 18.^1^ AGV almost always presents with acute abdominal pain, abdominal distention, and nonbilious emesis.^2^

Congenital diaphragmatic hernia (CDH) is an orifice in the diaphragm resulting from incomplete development of the pleuroperitoneal canal. It occurs in < 1–5:10000 births. Congenital diaphragmatic hernia is typically diagnosed during prenatal ultrasonography screening or within the first few days of life.^3^ We describe a previously healthy male infant with atypical presentation of acute gastric volvulus incarcerated in an undiagnosed congenital diaphragmatic hernia.

## Presenting concerns and clinical findings

A previously healthy six-month-old baby boy suddenly stopped breastfeeding after about one minute and started to arch his back and retch. The infant had no significant past medical history and no past surgeries. When his symptoms continued for 12 hours, his parents decided to visit the emergency department (ED) of the local hospital. They reported sudden onset abdominal distention and non-productive retching. On initial presentation in the ED, the patient seemed comfortable. Feeding and re-hydration with Pedialyte were attempted in the ED, but the patient continued to arch his back and retch. Plain abdominal X-ray revealed marked gaseous dilation of the stomach and minimal small bowel gas (yellow arrow). Complete blood count with differential showed elevated platelets and neutrophils. Comprehensive metabolic panel revealed acidosis with an elevated anion gap and hypoglycemia. He was fluid resuscitated with intravenous normal saline and then transferred to a tertiary pediatric center.

After transfer to the tertiary care center, the stomach was decompressed with a nasogastric tube, and 150 mL non-bilious fluid was drained. This maneuver yielded partial resolution of the abdominal distension.

## Significant findings

An upper gastrointestinal series (UGI) showed an enteric tube with its tip in the stomach and side-port in the esophagus. There was a large amount of air in the stomach and a small volume of scattered distal bowel gas. The tip of an enteric tube was seen in the stomach (red arrow). Contrast partially filled the stomach, and the greater curvature was visualized superior to the lesser curvature in the left upper quadrant (blue arrow). The body of the stomach was herniated into the right chest through a Bochdalek hernia (blue star). There was a large amount of air in the stomach and a small volume of scattered distal bowel gas. These findings were consistent with mesenteroaxial gastric volvulus.^4^

## Patient course

After 12 hours of retching on attempted feedings, this previously healthy six-month-old boy presented to the ED with abdominal distention, gaseous dilation of the stomach with minimal small bowel gas, anion gap metabolic acidosis, and hypoglycemia. At this time, the differential diagnosis included gastroenteritis, gastroesophageal reflux disease, esophagitis, foreign body ingestion, or pyloric stenosis.

After fluid resuscitation, the patient was transferred to a tertiary care center. In this ED, decompression with an NG tube yielded 150 mL non-bilious fluid and partial resolution of abdominal distention. UGI revealed mesenteroaxial gastric volvulus. Diagnosis of acute gastric volvulus indicates emergent surgery.^2^

Under general anesthesia, exploratory laparoscopy was performed. The stomach appeared massively distended with the fundus and body twisted and stuck in the upper left quadrant. Further inspection revealed a six-by-three-centimeter elliptical defect in the left posterior diaphragm. Both the stomach and spleen were entrapped in a diaphragmatic hernia sac.

In the operating room, the stomach was de-rotated and pexied to the anterior abdominal wall, and the diaphragmatic defect was closed. The patient tolerated the surgery well, and his recovery was smooth. Postoperative blood work revealed unremarkable electrolytes and complete blood cell count. On postoperative day one, X-ray showed a moderate-sized left-sided pneumothorax (green arrow). His nasogastric tube was removed on post-operative day two, and he tolerated breast milk from a bottle. Beginning on post-operative day three, he was breastfed without complications. On post-operative day three, the chest X-ray showed a minimal residual pneumothorax (green arrow), which sub-totally resolved the subsequent day (orange arrow). He was discharged on postoperative day four on a regular diet without need for pain medication. His final chest X-ray showed full pulmonary expansion without pneumothorax.

One month later, the patient continued to tolerate a normal diet, having regular bowel movements. X-ray on post operative day 33 showed resolution of gastric distention, volvulus, and pneumothorax. Normal bowel gas is seen.

The parents expressed gratitude for the swift responsiveness, systematic diagnostic approach, and high-quality care across the medical teams at both institutions. At his one-month postoperative visit, the patient was tolerating PO intake well, having regular bowel movements, and voiding appropriately. The mother was enthusiastic about the publication of this case so that it might be helpful to providers to identify patients like her son. Unfortunately, the family was lost to follow up six months postoperatively. The family was living in a rural area and frequently changed phone numbers and addresses. When the care team attempted to reach them six months postoperatively, their phone was disconnected, and letters mailed to their home address went unanswered. Improvements are needed in case management for rural families such as this one.

## Discussion

The reported case describes the coexistence of two rare surgical conditions: undiagnosed congenital diaphragmatic hernia (CDH) and atypical presentation of acute gastric volvulus (AGV).^5^ Concurrent CDH and AGV are likely related.^6^ Two of the four ligaments that normally anchor the stomach to prevent rotation, the gastrophrenic and gastrosplenic ligaments, anchor to the diaphragm. A defect in the left posterior diaphragm compromises the anchor point of these ligaments. This connection has also been discussed by Cribbs et. al in a review of pediatric gastric volvulus and demonstrated in a few case reports.^2^ Splenic herniation into a left-sided diaphragmatic defect is common and can lead to pre- and intraoperative complications.^7^ These cases demonstrate the importance of investigation of underlying CDH when AGV is identified.

The most common presentation of AGV in children is nonbilious emesis or retching without emesis.^2^ Gastric volvulus was first described by Berti in 1866. In 1904, Borchardt defined the Borchardt triad as the three defining signs of AGV: localized epigastric distention, retching, and inability to pass a nasogastric tube. In the past century, it has been shown that as few as 30% of adults with AGV present according to this criteria.^8^ This presentation is even less frequently seen in pediatric patients. Children under one year old almost always present with vomiting.^2^ The patient described here presented with generalized abdominal distension and non-productive retching. Retching without vomiting with abdominal distension is a clinical cue for gastric volvulus.^9^ This case contributes to a body of evidence that the Borchardt triad does not apply to all patients with AGV, especially pediatric patients. The team was able to pass a nasogastric tube to relieve some distention. It is important to report cases that deviate from postulated pathognomies, such as the Borchardt triad, to identify and adequately treat outliers. Given the emergent nature of this condition, a high index of suspicion is warranted to quickly initiate potentially life-saving diagnostics and treatment.

Upper gastrointestinal series (UGI) is a commonly accepted method for diagnosing AGV. Once identified, an attempt to advance a nasogastric tube should be made to relieve distention to the extent possible. Computed tomography (CT) scan provides a greater degree of sensitivity, with some studies reporting up to 100% sensitivity.^10^,^11^ In this case, the herniation of the stomach into the chest was missed in the read of the UGI, while it would have been almost certainly identified by CT. However, CT would have delayed treatment and exposed the child to more radiation, and it would not have changed the indicated surgical intervention. In emergent cases such as AGV, treatment should not be delayed for diagnostic tests with greater sensitivity if the diagnostic information would not meaningfully inform the treatment course.

Nonoperative management in pediatric cases of AGV has an 80% mortality rate, and delay of diagnosis increases the risk of morbidity and mortality. Due to impingement on the vascularity of the gastric tissue, ischemia and necrosis of the gastric wall may occur. This could lead to perforation, peritonitis, or sepsis.^8^ If the degree of necrosis of gastric wall presents, a partial or complete gastrectomy may be required. Chronic or intermittent gastric volvulus may also present with failure to thrive and with possible lethal consequences.^4^ An additional dire consequence of undiagnosed or untreated AGV is gastric strangulation.^8^

Preferred surgical treatment for AGV includes laparoscopic or open de-rotation of the volvulus and gastropexy to the anterior abdominal wall. The latter prevents a recurrent volvulus. As many as 70% of AGV cases are the result an underlying medical cause.^8^ As this case illustrates, underlying causes should be investigated, identified, and treated. This case is a demonstration of swift action by teams at two medical facilities to appropriately diagnose and treat a rare presentation of two rare but likely related conditions in a six-month-old. The primary limitation is that the family was lost to follow-up, exposing the need for better case management for rural patients and families.

Acute gastric volvulus is a surgical emergency that warrants a high index of suspicion, swift response, and investigation of associated hernia. Emergency medicine physicians should consider it in the differential diagnosis if a child presents with abdominal discomfort, abdominal distention, retching with or without non-bilious emesis, failure to thrive, or inability to pass a nasogastric tube. Plain radiography is the initial diagnostic test, and it will show one large gas sphere in the upper abdomen or chest with an air-fluid level.^4^ If plain radiography is inconclusive but AGV is suspected, upper GI series is the gold standard in pediatric patients to avoid excessive radiation from CT imaging.^2^ If imaging reveals AGV, emergency surgical consultation is necessary.

**Figure:**
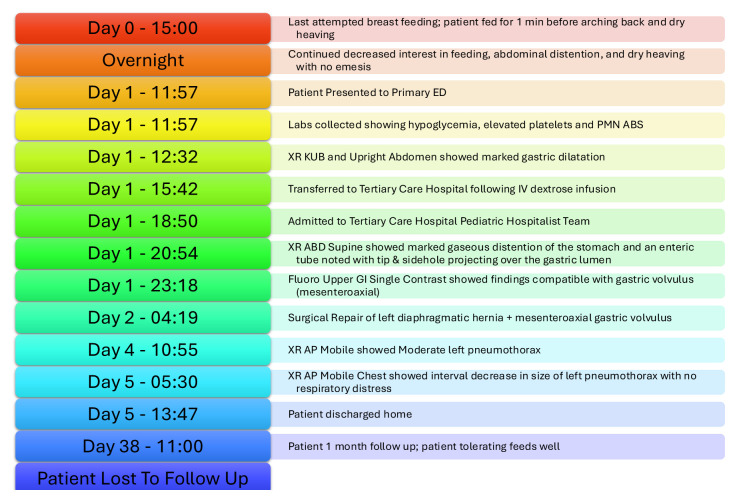


## Supplementary Information


















