# Supplementary figures and images for: Case Report of Incarcerated Gastric Volvulus and Splenic Herniation in Undiagnosed Congenital Diaphragmatic Hernia in an Infant
Source: J Educ Teach Emerg Med. 2025 Jul 31;10(3):V16–21. doi: 10.21980/J8VD27 (PMC12320996; doi:10.21980/J8VD27)

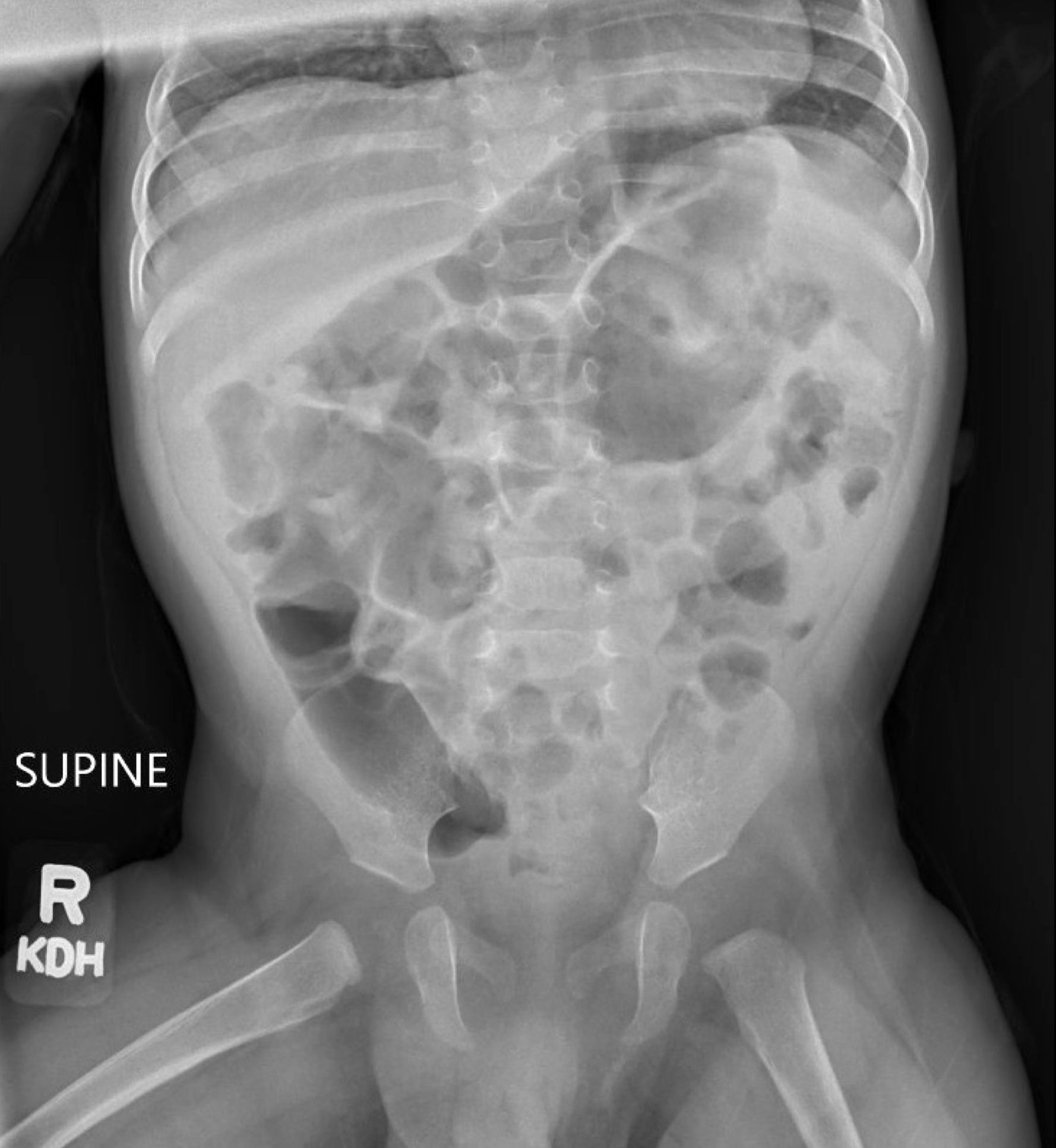

Supplement: Supplementary file 1 [file 10-3-V16-Supp1.jpg]

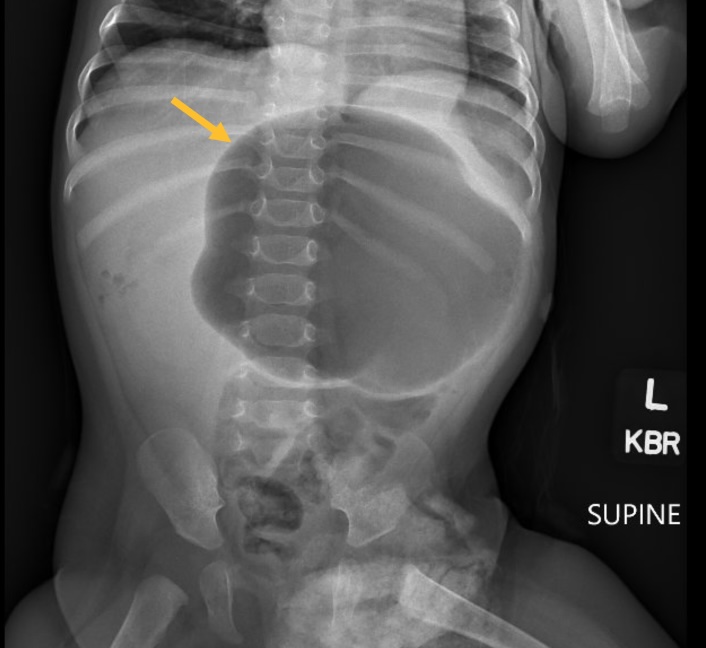

Supplement: Supplementary file 2 [file 10-3-V16-Supp2.jpg]

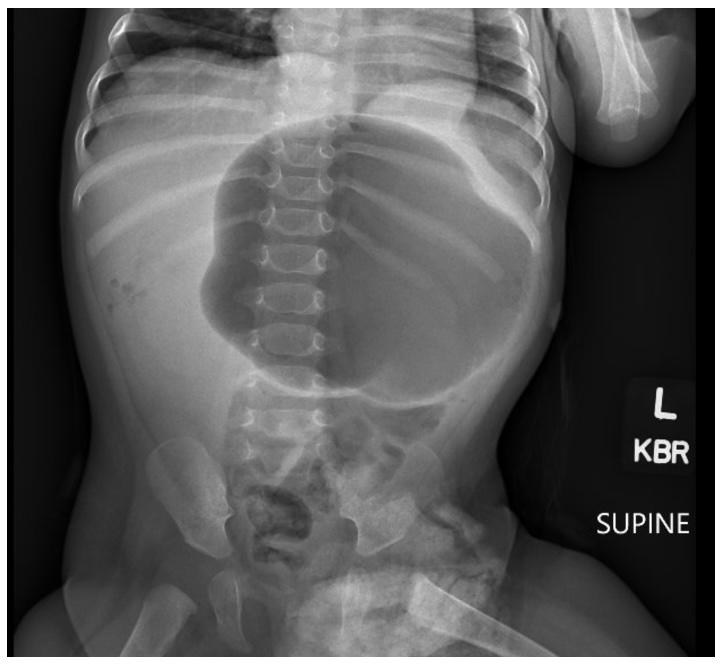

Supplement: Supplementary file 3 [file 10-3-V16-Supp3.jpg]

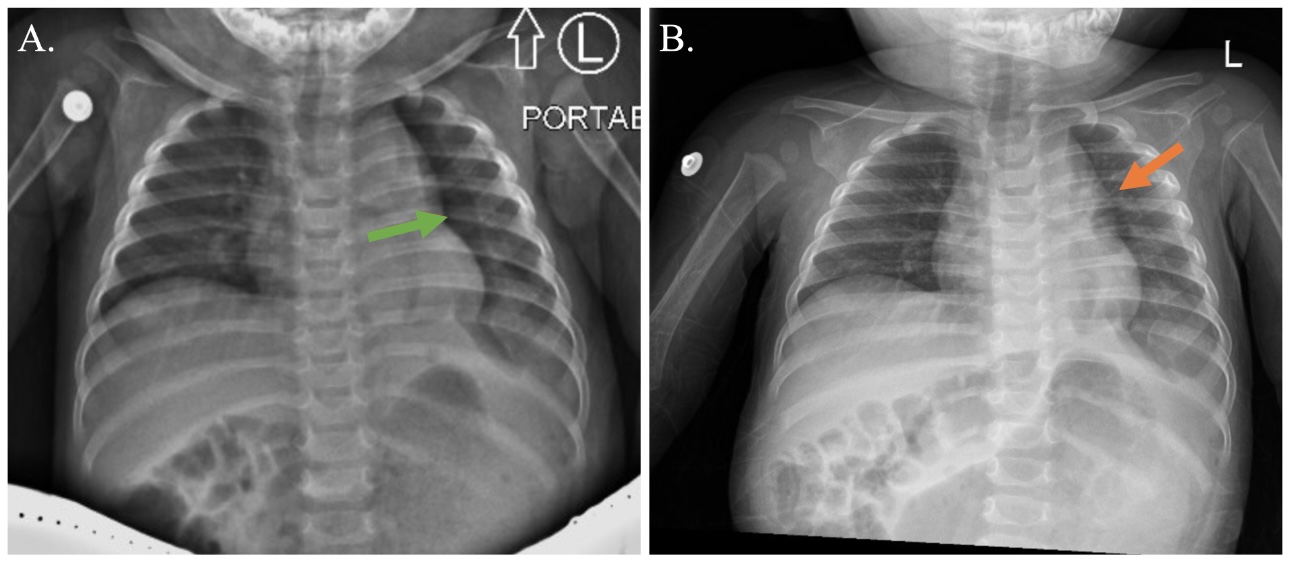

Supplement: Supplementary file 4 [file 10-3-V16-Supp4.jpg]

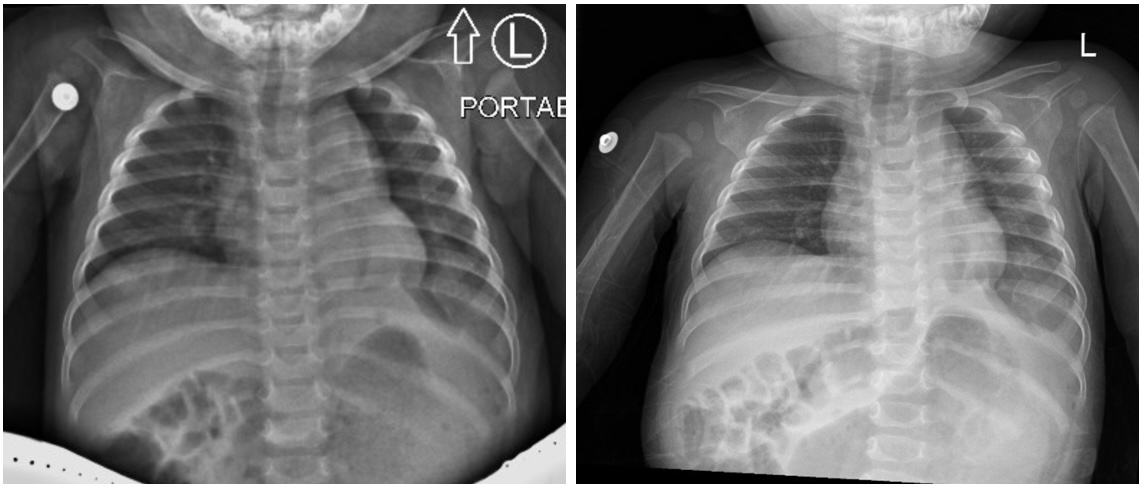

Supplement: Supplementary file 5 [file 10-3-V16-Supp5.jpg]

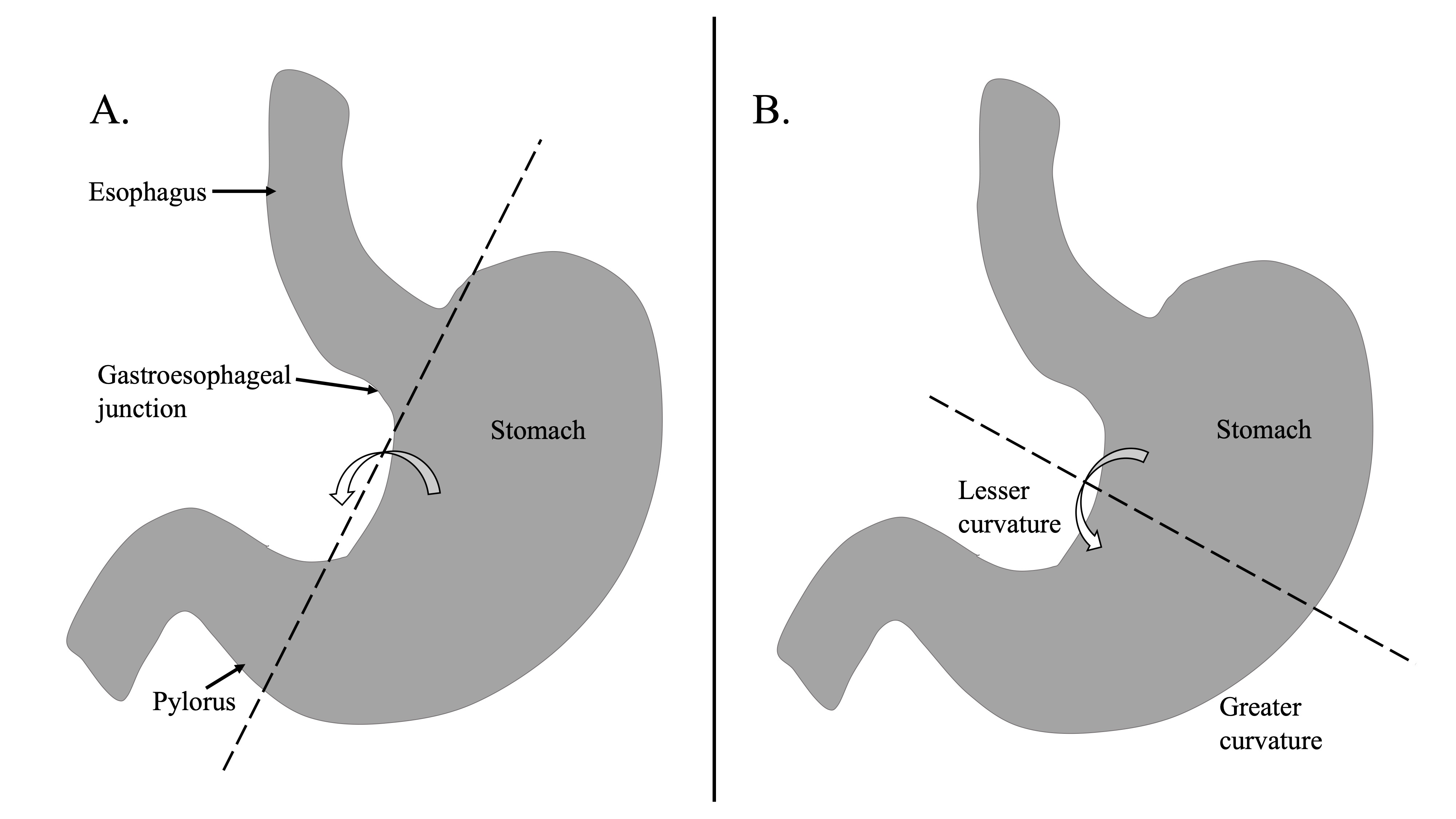

Supplement: Supplementary file 6 [file 10-3-V16-Supp6.jpg]

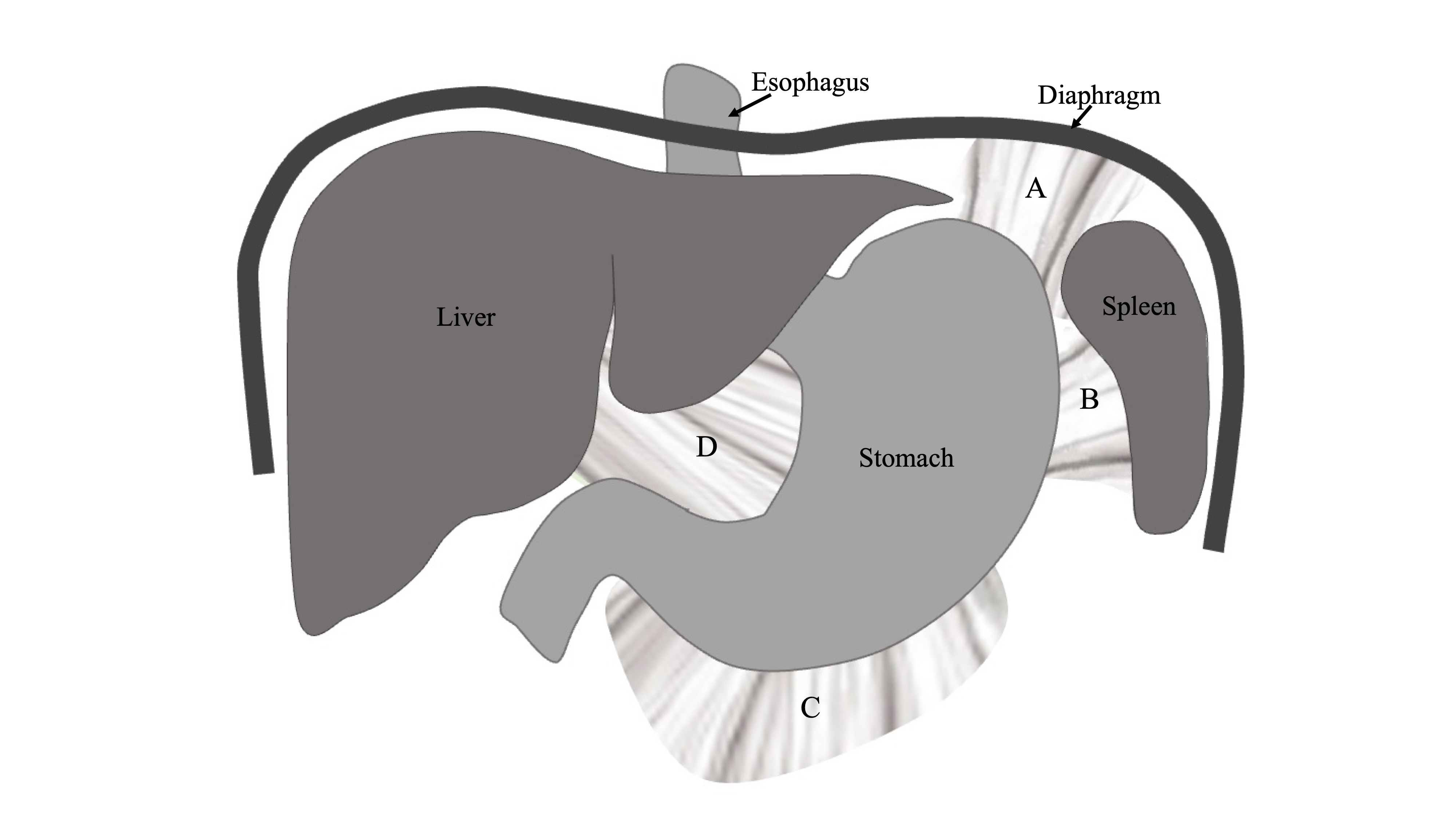

Supplement: Supplementary file 7 [file 10-3-V16-Supp7.jpg]

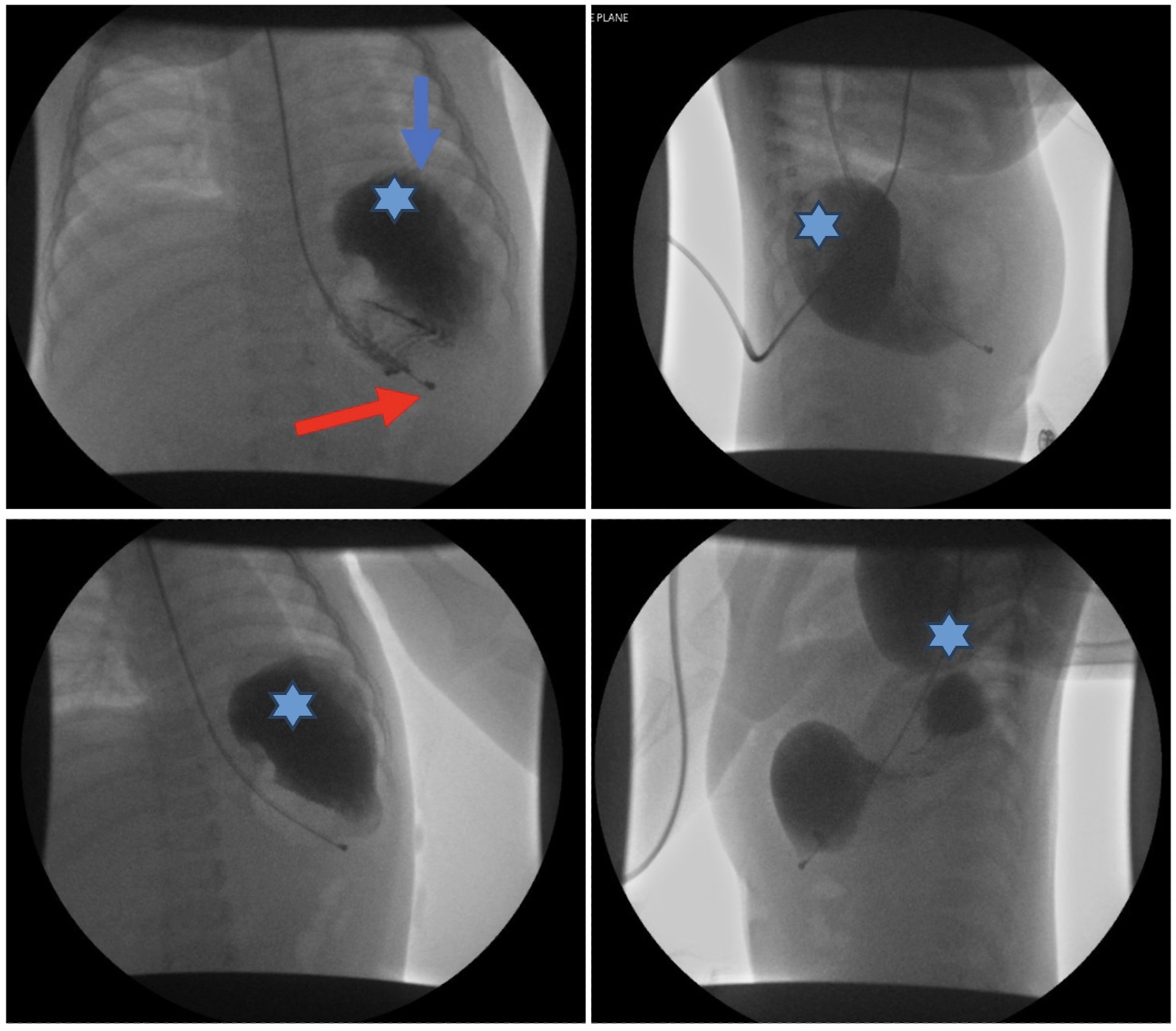

Supplement: Supplementary file 8 [file 10-3-V16-Supp8.jpg]

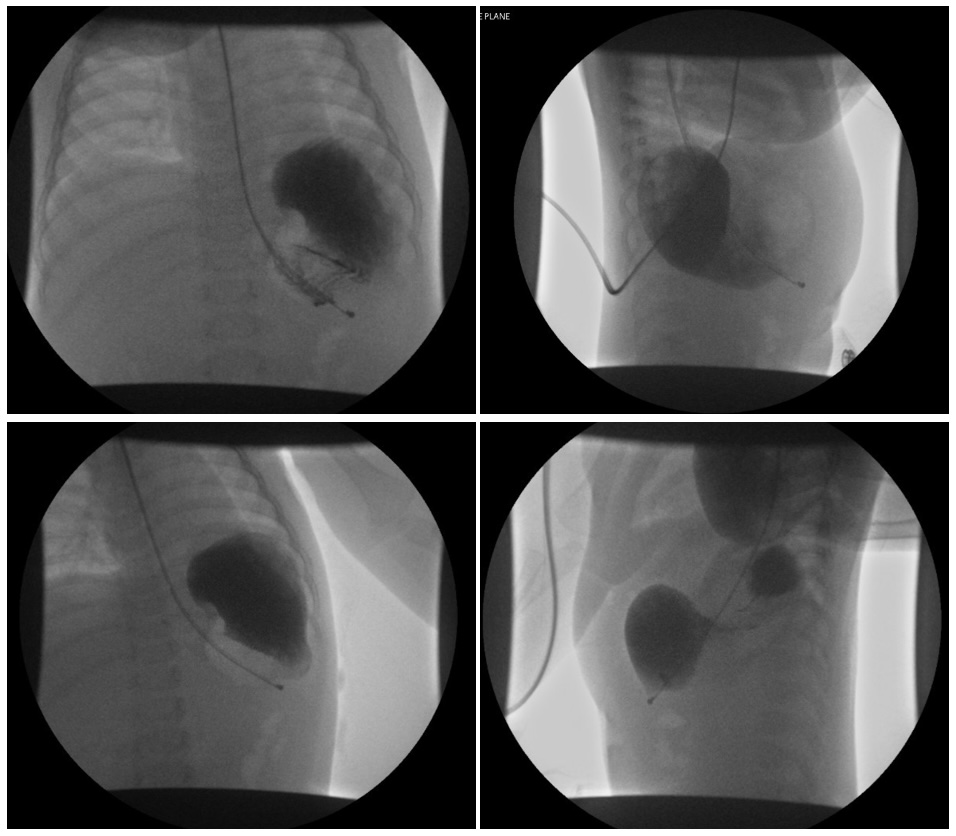

Supplement: Supplementary file 9 [file 10-3-V16-Supp9.jpg]
